# Financial toxicity and patient experience associated with financial burden of molecular-targeted and immune therapies for cancer: an observational study under public health insurance

**DOI:** 10.1007/s10147-024-02479-2

**Published:** 2024-02-24

**Authors:** Sena Yamamoto, Chiharu Kondoh, Hideko Nakagoshi, Mayuko Kakumen, Kana Yasuhara, Mayumi Nakai, Naoko Kodani, Kazumi Sunda, Chizuru Higashide, Megumi Katayama, Harue Arao

**Affiliations:** 1 Osaka University Graduate School of Medicine, Division of Health Sciences, 1-7 Yamadaoka, Suita, Osaka 565-0871 Japan; 2 Sumitomo Hospital, 5-3-20 Nakanoshima Kita-Ku, Osaka, Osaka 535-0005 Japan; 3 Nursing Department, Hyogo Prefectural Amagasaki General Medical Center, 2-17-77 Higashinaniwa-Cho, Amagasaki, Hyogo 660-8550 Japan; 4 Matsushita Memorial Hospital, 5-55 Sotojima-Cho, Moriguchi, Osaka 570-8540 Japan; 5 National Hospital Organization Osaka National Hospital, 2-1-14 Hoenzaka, Chuo-Ku Osaka, Osaka, 540-0006 Japan; 6 Department of Nursing, National Hospital Organization Nagoya Medical Center, 4-1-1, Sannomaru, Naka-Ku, Nagoya, Aichi 460-0001 Japan; 7 National Hospital Organization, Yonago Medical Center, 4-17-1 Kuzumo, Yonago, Tottori 683-0006 Japan; 8 Social Welfare Organization Imperial Gift Foundation, Inc. Saiseikai Shiga Hospital, 2-4-1 Ohashi, Ritto, Shiga 520-3046 Japan; 9 Shiga General Hospital, 5-4-30 Moriyama, Moriyama, Shiga 524-8524 Japan; 10 Kyoto Chubu Medical Center, 25 Yagi-Cho Yagi Ueno, Nantan, Kyoto 629-0197 Japan

**Keywords:** Cancer, Financial toxicity, Molecular targeted therapy, Immunotherapy

## Abstract

**Background:**

Financial burden of cancer treatment can negatively affect patients and their families. This study aimed to evaluate the financial toxicity of patients treated with molecular-targeted and immune therapies and explore the relationship between financial toxicity and patient experiences associated with the financial burden of cancer treatment.

**Methods:**

This anonymous, self-administered questionnaire survey conducted across nine hospitals in Japan included patients aged 20–60 years who were receiving molecular-targeted agents or immune checkpoint inhibitors for any type of cancer for ≥ 2 months. Financial toxicity was evaluated using the COmprehensive Score for Financial Toxicity (COST). Patient experience was examined using 11 items based on previous studies. Independent factors related to financial toxicity were explored using multiple regression analyses.

**Results:**

The mean COST score was 17.0 ± 8.4, and 68 (49.3%) participants reported COST scores at or below the cutoff point. The factors contributing to financial toxicity were “hesitation regarding continuing treatment based on finances” (sβ = − 0.410, p < 0.001), “cutting through my deposits and savings” (sβ = − 0.253, p = 0.003), and “reducing spending on basics like food or clothing” (sβ = − 0.205, p = 0.046) along with comorbidities (sβ = − 0.156, p = 0.032).

**Conclusion:**

Patients receiving molecular-targeted and immune therapies are at risk of experiencing profound financial toxicity and a reduced quality of life. The independently related factors that we identified have the potential to serve as indicators of profound financial toxicity and the need for specialized intervention.

## Introduction

A greater understanding of the molecular and immunologic processes driving cancer contributes to continued innovation of cancer treatment, while rising costs of the treatment has the potential to increase the risk of financial hardship [1]. Financial toxicity (FT) is a term proposed to describe the impact of the cost of cancer care at patient-level [2], referring to a potential consequence of subjective financial distress that patients experience owing to cancer-related treatment costs [3]. FT is a unique term used to consider financial complaints as being of the same nature as physical toxicity [2]. FT was reported in 28–48% of cancer survivors using monetary measures and in 16–73% of cancer survivors using subjective measures [4]. Molecular-targeted drugs and immune checkpoint inhibitors have been identified as risk factors for FT [5]; however, FT due to molecular-targeted and immune therapies has not been thoroughly explored. The impact of FT is diverse and includes personal-related outcomes, such as lifestyle changes, poor quality of life, and bankruptcy, as well as disease-related outcomes, such as the abandonment, delay, and discontinuation of treatment and, in serious cases, an increased risk of mortality [6]. Even in publicly funded health care systems, FT can potentially lead to negative outcomes for patients with cancer [7]. National health insurance system in Japan reduces out-of-pocket expenses for insured medical care to 30% of the total treatment cost or 10–20%, depending on specific conditions including patients’ age and income. In addition, the High-Cost Medical Expense Benefit System further reduces monthly out-of-pocket expenses when utilized. Despite these mitigating systems, FT is a substantial issue in Japan [8, 9]. A systematic review supports the notion that public healthcare insurance does not necessarily protect against FT [10].

Patient-physician discussions about costs were limited to 24% in oncology settings, even when broadening the definition of cost conversation to its most liberal interpretation, and the rate of cost conversations was lower than that in non-oncology settings [11]. Despite the fact that 52% of patients with cancer wanted to talk to their oncologist about treatment-related costs, only 19% actually did [12]. Only half of the oncologists were aware of the subjective burden their patients experienced, which strongly suggests that oncology professionals underestimate the subjective financial burden [13]. For oncology professionals to detect FT in cancer care before FT gets worse, they should pay closer attention to patient experiences to find clues. The impacts of financial hardship on patients with cancer and their families appear on various domains, including family finances, lifestyle, psychological well-being, health, spiritual well-being, and family dynamics [14]. Parallelly, patients facing FT try to address financial burden using mitigating strategies such as making financial and emotional adjustments and seeking support and resources to cope [15]. The relationship between FT and patient experience is also theoretically consistent with the framework that objective financial burden manifests as FT through subjective financial distress [3]. Turning to patient experiences could aid in enhancing our sensitivity to FT and referrals to appropriate financial resources.

Therefore, this study aimed to evaluate the FT of patients with cancer, undergoing outpatient chemotherapy with molecular-targeted drugs and immune checkpoint inhibitors, and to explore the independent factors related to FT for clinical assessment.

## Patients and methods

### Patients

We recruited cancer outpatients from nine hospitals in Japan. Inclusion criteria included continuing a regimen containing any molecular-targeted agent or immune checkpoint inhibitor as cancer treatment for ≥ 2 months (combination of cytotoxic therapy was not considered), being informed of his/her cancer diagnosis, being 20–60 years of age at the time of the survey request, and being able to fill out the questionnaire independently. The age limit was set because Japanese people usually experience change in income due to retirement (and being rehired in some cases) at the age of 60, and people over the age of 70 who meet the requirements pay less out-of-pocket treatment expenses owing to medical care system for older individuals. Exclusion criteria included receiving welfare benefits at the time of the survey request and receiving each dose of medication under inpatient care.

### Procedures for data collection

An anonymous, self-administered questionnaire was administered from October 2021 to August 2022. This study was approved by the Ethical Review Board of Osaka University Hospital (No. 21267 (T7)) and the participating hospitals. Nominated clinical nurses from each hospital selected research candidates in a convenient way and briefed them on the study. Candidates gave verbal consent prior to their inclusion. The included participants were given questionnaire forms along with a return envelope addressed to the institution of the research representative, were instructed to fill out the questionnaire themselves, and provided a checkbox to express their consent at the beginning of the questionnaire. The returned questionnaire with an entry in this consent section was considered as consent to participate.

### Measurements

#### Financial toxicity

FT was evaluated using the Japanese translation of the COmprehensive Score for Financial Toxicity (COST) [8, 9]. The original version of the COST [16] was developed to assess FT in patients with cancer, and its reliability and validity have been confirmed [17]. The COST is an 11-item scale, and respondents rate each item for the previous week on a 5-point scale ranging from 0 (not at all) to 4 (very much). After inverting the scores of the reversed items, a total score between 0 and 44 points is calculated. A lower score indicates greater FT. A previous study proposed a cutoff of 17.5 point to predict an adverse outcome for quality of life (sensitivity, 0.673; specificity, 0.657) [5].

#### Experience associated with the financial burden of cancer treatment

Based on a literature review and the nationwide Cancer Patient Experience Survey in Japan, 11 items were extracted [3, 9, 15, 18, 19]. Respondents were asked whether they had experienced each item since starting the cancer treatment they were receiving. If respondents had experienced the items, they rated the degree of their distress on a 3-point scale (0 = not distressed; 1 = distressed; 2 = strongly distressed).

#### Patient characteristics and information related to financial status

Demographic and clinical data regarding patient characteristics were collected using the questionnaire. We also collected information on expenditures (such as out-of-pocket treatment expenses, frequency of hospital visits for cancer treatment, and subscription to private health insurance) and information related to income (such as employment status, household income, and household savings).

### Statistical analysis

All variables were summarized using descriptive statistics. Then, using t-tests, we first compared the COST score according to whether the participants had experienced each item. Second, the correlation between the COST score and the number of items that the participants had experienced was investigated. Third, we compared the COST score by participant characteristics. Correlation coefficients were calculated for continuous variables, and *p* values were calculated using t-tests (two groups) and one-way analysis of variance (ANOVA) (three or more groups) for categorical variables. When differences among groups were significant in ANOVA, Tukey post-hoc tests or Games–Howell post-hoc tests were conducted according to whether or not equal variances were assumed. Finally, a multiple regression analysis was conducted to explore independent factors associated with a lower COST score. The items that achieved *p* < 0.05 in univariable analysis were entered by the forward selection method. The significance level was defined as *p* < 0.05. IBM SPSS statistics, version 28.0 (IBM Japan Ltd., Tokyo, Japan), was used for statistical analysis.

## Results

The 174 copies of the questionnaire were distributed to research participants. A total of 149 (85.6%) copies were returned, of which 138 (79.3%) were analyzed. The 11 copies that were excluded were six cases of respondents not meeting the inclusion criteria (ineligible age), three cases that had extremely missing values on the COST measure (none of, or only 1 item responded), and two cases that did not give consent.

### Summary of participants

Table 1 shows the participant’s characteristics. Participants were diagnosed with cancer at a mean age of 48.3 ± 7.3 years (mean ± standard deviation), and their mean age at the time of the survey was 51.5 ± 7.1 years. The majority of participants were female (69.6%). Breast cancer was the most common type (43.5%). Most participants received molecular-targeted therapy (71.7%) and continued the treatment without determining its duration (typically, metastatic or recurrent cancer treatment) (57.2%).

**Table 1:** Clinical and demographic characteristics of the participants (N = 138)

Characteristic		n	%
Age at time of response (years)		51.5 ± 7.1	
Age at cancer diagnosis (years)		48.3 ± 7.3	
Gender	Female	96	69.6
	Male	40	29.0
Primary tumor site	Breast	60	43.5
	Colon/rectum	23	16.7
	Lung	17	12.3
	Lymph node	8	5.8
	Ovary	7	5.1
	Stomach	6	4.3
	Other	16	11.6
Type of current treatment	Molecular targeted therapy	99	71.7
	Immunotherapy	20	14.5
	Combined	1	0.7
	Unknown/no answer	18	13.0
Duration of current treatment at the time of response (months)		11 (4–19)	
Planned duration of current treatment	Specifically determined	52	37.7
	Continue without determining	79	57.2
	Unknown	5	3.6
History of cancer treatment	Yes	80	58.0
	No	55	39.9
Comorbidities	Yes	37	26.8
	No	98	71.0
Marital status	Married/common-law marriage	100	72.5
	Unmarried	28	20.3
	Separation/bereavement	10	7.2
Household size		3 (2–4)	
Education level	Junior high school	1	0.7
	High school	56	40.6
	Junior college/professional training college	38	27.5
	University/graduate school	43	31.2

Values are presented as the mean ± standard deviation or median (interquartile range)

Due to missing numbers, the total may not add up to 100%

**Fig. 1:**
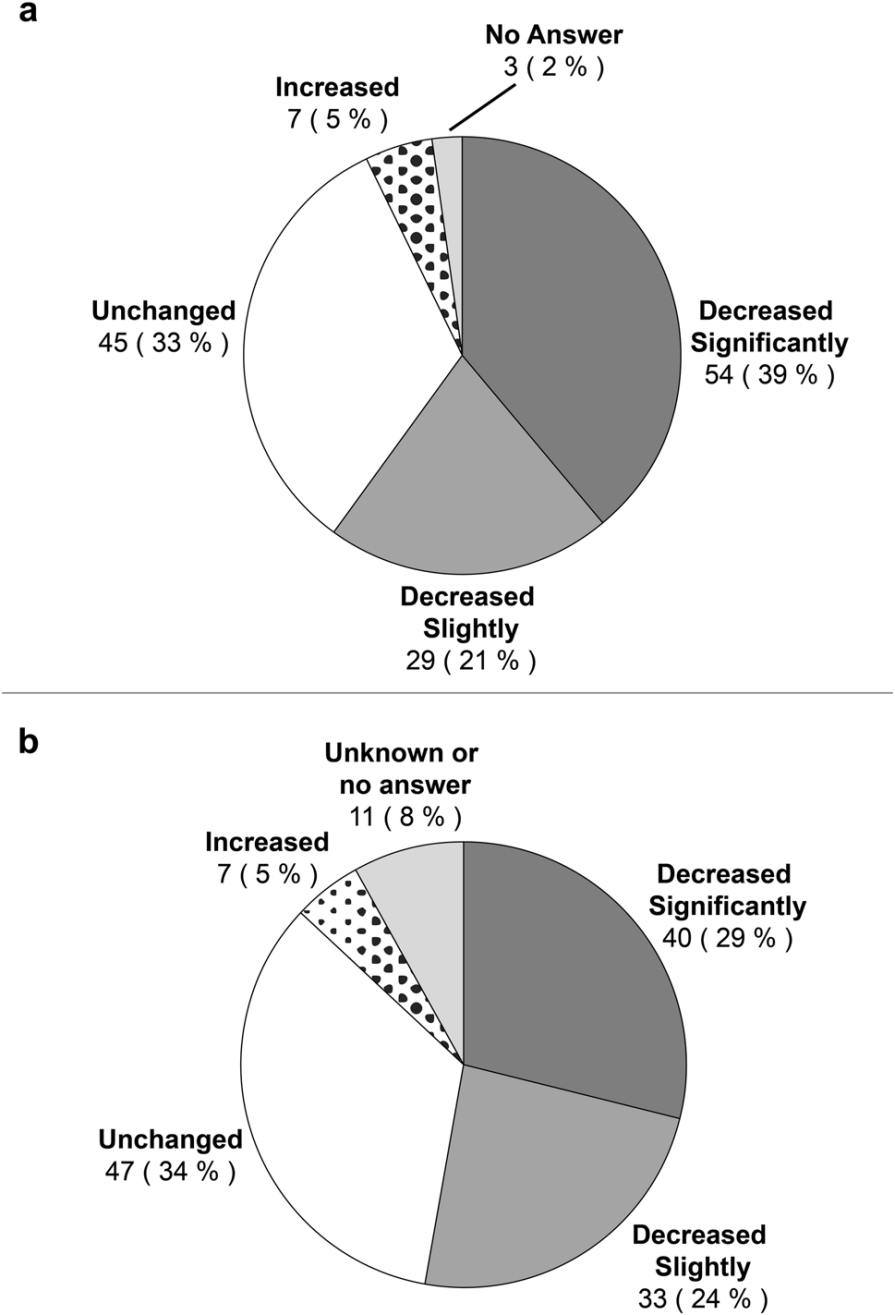
Changes in **a** personal income and **b** household income after cancer diagnosis

Details of the financial status of the participants are shown in Table 2. Approximately half (51.4%) of the participants paid 40,000–60,000 JPY per month on average over the prior 2 months. Subscription to private insurance was widespread (84.1%). A total of 86 (62.3%) participants were employed, but 30 (21.7%) had irregular employment. Figure 1 shows changes in income after a cancer diagnosis. The personal income after cancer diagnosis “decreased significantly” in 54 participants (39.1%) and “decreased slightly” in 29 participants (21.0%), indicating that income had decreased in 60.1% of the participants. Furthermore, 73 participants (52.9%) reported a decrease in household income as well.

**Table 2:** Financial status of the participants (N = 138)

Variable		n	%
Mean out-of-pocket treatment expenses per month over the last 2 months (JPY)	Less than 20,000	0	0.0
	20,000 to 40,000	16	11.6
	40,000 to 60,000	71	51.4
	60,000 to 80,000	9	6.5
	80,000 to 100,000	9	6.5
	100,000 or more	28	20.3
Frequency of hospital visits for cancer treatment	Once a week or more	15	10.9
	About once in 2 weeks	34	24.6
	About once in 3 weeks	63	45.7
	About once a month	15	10.9
	Other	10	7.2
Subscription to private insurance	Yes	116	84.1
	No	21	15.2
Employment status	Employed		
	Regular	45	32.6
	Irregular	30	21.7
	Self-employed	11	8.0
	On leave	20	14.5
	Retired (due to cancer)	14	10.1
	Retired (other reasons)	3	2.2
	Housewife	11	8.0
	Other	4	2.9
Annual household income (JPY)	Less than 4 million	34	24.6
	4 million to 6 million	37	26.8
	6 million to 8 million	22	15.9
	8 million to 10 million	11	8.0
	10 million or more	21	15.2
	Refusal to answer/unknown	12	8.7
Household savings (JPY)	Less than 2 million	32	23.2
	2 million to 4 million	16	11.6
	4 million to 6 million	12	8.7
	6 million to 8 million	11	8.0
	8 million to 10 million	9	6.5
	10 million or more	27	19.6
	Refusal to answer/unknown	30	21.7

Due to missing numbers, the total may not add up to 100%

### Financial toxicity based on the COST measure

Table 3 shows the COST score that the participants reported. Cronbach’s alpha for the COST score was 0.850. The mean and median total COST scores of the participants were 17.0 ± 8.4 points and 18 points, respectively. A total of 68 (49.3%) participants reported COST scores at the cutoff point or lower. The items with a low mean score in ascending order were “I know that I have enough money in savings, retirement, or assets to cover the costs of my treatment” (0.8 ± 1.0), “I am satisfied with my current financial situation” (0.9 ± 0.9), and “I feel in control of my financial situation” (1.3 ± 1.1).

**Table 3:** Financial toxicity evaluated using the COmprehensive Score for Financial Toxicity (N = 138)

	Mean ± SD	Median
Total scores of the COST measure	17.0 ± 8.4	18

	n	%
High level of financial toxicity (COST ≤ 17.5)	68	49.3
Low level of financial toxicity (COST > 17.5)	70	50.7

*COST* COmprehensive Score for Financial Toxicity

### Experience associated with the financial burden of cancer treatment

In terms of frequency of experience, “thinking about how to make ends meet” was the most frequently experienced (99 participants, 71.7%). Although 55 participants (39.9%) experienced “hesitation regarding continuing treatment based on finances,” only 25 participants (18.1%) had the experience of “talking to health care providers about financial worries.” The most common “strongly distressed” experience was “relying on relatives and others to help with the costs of health care” (10/17, 58.8%), followed by “cutting through my deposits and savings” (38/80, 47.5%) and “hesitation regarding continuing treatment based on finances” (24/55, 43.6%).

### Relationship between the COST score and the experience associated with the financial burden of cancer treatment

**Fig. 2:**
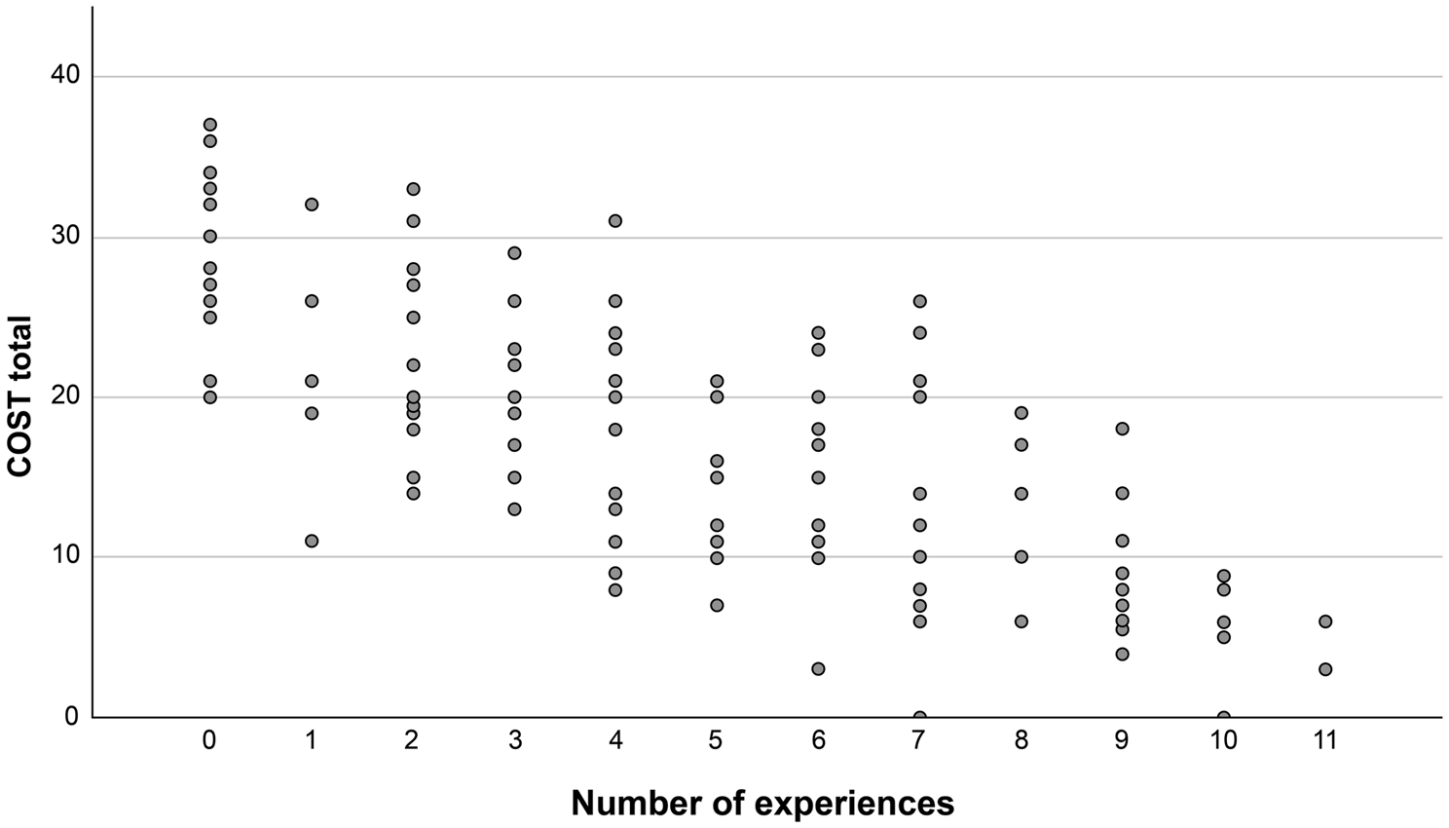
Correlation between financial toxicity and the accumulation of experiences associated with the financial burden of cancer treatment (n = 134) Pearson correlation coefficient, *r* =  − 0.715, *p* < 0.001. The vertical axis depicts scores of the COmprehensive Score for Financial Toxicity (COST), and the horizontal axis depicts the accumulated number of items responded to that participants have experienced

Table 4 compares the COST scores according to prior experience associated with the financial burden of cancer treatment. The COST scores were significantly lower when prior experiences were present compared to when there was no prior experience for all items, except for “taking measures for using public subsidies and support systems.” As shown in Fig. 2, the number of items that the participants experienced was significantly correlated to the COST scores (*r* = − 0.715, *p* < 0.001); that is, the COST scores significantly worsened as these experiences accumulated.

**Table 4:** Differences in the COmprehensive Score for Financial Toxicity by prior experience associated with the financial burden of cancer treatment (N = 138)

		n	Mean ± SD	*p*
Thinking about how to make ends meet	Experienced	99	14.3 ± 7.3	< 0.001
	Not experienced	39	23.9 ± 7.0	
Making my family and those around me financially worried	Experienced	86	13.9 ± 7.0	< 0.001
	Not experienced	51	22.4 ± 8.1	
Taking measures for using public subsidies and support systems	Experienced	85	16.2 ± 7.7	0.134
	Not experienced	53	18.4 ± 9.4	
Cutting through my deposits and savings	Experienced	80	13.3 ± 6.9	< 0.001
	Not experienced	57	22.3 ± 7.5	
Reducing leisure activities more than usual	Experienced	78	13.8 ± 6.9	< 0.001
	Not experienced	60	21.2 ± 8.4	
Reducing spending on basics like food or clothing	Experienced	64	11.5 ± 6.6	< 0.001
	Not experienced	73	21.8 ± 6.7	
Forcing myself to work to earn an income or increasing work burden for family	Experienced	64	13.4 ± 6.7	< 0.001
	Not experienced	74	20.2 ± 8.5	
Hesitation regarding continuing treatment based on finances	Experienced	55	10.8 ± 6.5	< 0.001
	Not experienced	83	21.1 ± 6.9	
Adjusting medical examination schedules, tests, and treatment contents with financial circumstances	Experienced	25	9.5 ± 6.2	< 0.001
	Not experienced	112	18.8 ± 7.9	
Talking to health care providers about financial worries	Experienced	25	10.7 ± 6.8	< 0.001
	Not experienced	113	18.4 ± 8.1	
Relying on relatives and others to help with the costs of health care	Experienced	17	11.5 ± 6.4	0.004
	Not experienced	121	17.8 ± 8.4	

*SD* standard deviation

T-tests were used for analysis

### Relationship between the COST score and patient characteristics

Table 5 compares the COST scores by participant characteristics. Regarding clinical and demographic characteristics, the COST scores were significantly lower when participants had comorbidities (*p* = 0.020) and graduated from junior high school and high school (ANOVA, *p* < 0.001; Games–Howell post-hoc test, compared to university and graduate school, *p* < 0.001). Financial status of the participants including mean out-of-pocket treatment expenses per month (*p* = 0.049), employment status (*p* = 0.009), annual household income (*p* < 0.001), and household savings (*p* < 0.001) were also significantly correlated to the COST scores. Tukey post-hoc tests indicated that lower COST scores were significantly related to having paid 60,000 JPY or more (compared to 20,000 to 40,000 JPY, *p* = 0.045), being on leave or retired due to cancer (compared to employed, *p* = 0.007), having less than 4 million JPY as annual household income (compared to 4 million to 8 million, *p* = 0.004; to 8 million or more,* p* < 0.001), and having less than 4 million in household savings (compared to 4 million to 8 million, *p* = 0.048; to 8 million or more, *p* = 0.001).

**Table 5:** Differences in the COmprehensive Score for Financial Toxicity by participant characteristics (N = 138)

		n	*r*	*p*
Age at time of response (years)		136	− 0.007	0.936
Age at cancer diagnosis (years)		136	0.046	0.592

		n	Mean ± SD	*p*
Gender	Female	96	16.8 ± 8.7	0.607^a^
	Male	40	17.6 ± 7.7	
Type of current treatment	Molecular targeted therapy	99	16.5 ± 8.5	0.346^a^
	Immunotherapy	20	18.4 ± 8.0	
Duration of current treatment at the time of response (months)	Less than 12 months	69	18.0 ± 9.1	0.120^a^
	12 months or more	66	15.8 ± 7.7	
Planned duration of current treatment	Specifically determined	52	17.9 ± 7.8	0.417^a^
	Continue without determining	79	16.6 ± 9.0	
History of cancer treatment	Yes	80	16.7 ± 8.7	0.587^a^
	No	55	17.5 ± 8.1	
Comorbidities	Yes	37	14.1 ± 7.3	0.020^a^
	No	98	17.9 ± 8.6	
Marital status	Married/common-law marriage	100	16.9 ± 8.3	0.450^b^
	Unmarried	28	18.2 ± 8.8	
	Separation/bereavement	10	14.3 ± 8.3	
Household size	Two or more	120	17.0 ± 8.4	0.957^a^
	One only (alone)	18	17.1 ± 8.6	
Education level	Junior high school/High school	57	14.3 ± 9.3	< 0.001^b^
	Junior college/professional training college	38	17.3 ± 8.0	
	University/graduate school	43	20.4 ± 6.0	
Mean out-of-pocket treatment expenses per month over the last 2 months (JPY)	20,000 to 40,000	16	21.8 ± 9.2	0.049^b^
	40,000 to 60,000	71	16.6 ± 8.0	
	60,000 or more	46	15.9 ± 8.4	
Frequency of hospital visits for cancer treatment	Once a week or more	15	17.2 ± 11.2	0.618^b^
	About once in 2 weeks	34	16.9 ± 7.4	
	About once in 3 weeks	63	18.2 ± 7.9	
	About once a month	15	15.2 ± 7.8	
Subscription to private insurance	Yes	116	17.3 ± 8.5	0.216^a^
	No	21	14.8 ± 7.1	
Employment status	Employed	86	18.6 ± 8.3	0.009^b^
	On leave or retired (due to cancer)	34	13.5 ± 7.9	
	Others	18	15.9 ± 8.1	
Annual household income (JPY)	Less than 4 million	34	12.4 ± 6.3	< 0.001^b^
	4 million to 8 million	59	17.9 ± 8.1	
	8 million or more	32	21.5 ± 8.2	
Household savings (JPY)	Less than 4 million	48	14.0 ± 7.9	0.001^b^
	4 million to 8 million	23	18.7 ± 7.0	
	8 million or more	36	20.2 ± 8.1	

*SD* standard deviation

^a^t-tests

^b^one-way analysis of variance

### Factors affecting the COST scores in multiple regression analysis

Table 6 shows the results of multiple regression analysis with the COST score as the dependent variable. Factors independently related to the COST score were “hesitation regarding continuing treatment based on finances” (sβ = − 0.410, *p* < 0.001), “cutting through my deposits and savings” (sβ = − 0.253, *p* = 0.003), and “reducing spending on basics like food or clothing” (sβ = − 0.205, *p* = 0.046) in prior experience associated with the financial burden of cancer treatment, and having comorbidities (sβ = -0.156, *p* = 0.032) as clinical characteristics (analysis of variance, *p* < 0.001; adjusted R^2^ = 0.545; Durbin–Watson ratio = 1.962). The variance inflation factor ranged from 1.059 to 2.130.

**Table 6:** Independent factors related to the COmprehensive Score for Financial Toxicity (n = 95)

	B	95% confidence interval	sβ	*p*
Hesitation regarding continuing treatment based on finances^a^	− 6.871	− 9.903 to − 3.840	− 0.410	< 0.001
Cutting through my deposits and savings^a^	− 4.245	− 7.025 to − 1.464	− 0.253	0.003
Comorbidities^b^	− 3.003	− 5.747 to − 0.259	− 0.156	0.032
Reducing spending on basics like food or clothing^a^	− 3.405	− 6.753 to − 0.056	− 0.205	0.046
R^2^				0.565
Adjusted R^2^				0.545

Results are from multiple regression analysis entering the variables that achieved *p* < 0.05 in univariable analysis

^a^1 = experienced; 0 = not experienced

^b^1 = yes; 0 = no

## Discussion

To the best of our knowledge, this is the first study to evaluate the relationship between FT and patient experiences associated with financial burden of molecular-targeted and immune therapies. The main findings of this study are as follows: (1) one in two participants experienced a high level of FT that might have reduced their quality of life; (2) coping strategies to raise funds for treatment expenses was significantly related to higher FT; and (3) participants with higher FT hesitated more in continuing treatment based on finances.

The most important finding was observing what experiences were independently related to profound FT. Participants with higher FT attempted to raise funds for treatment expenses by cutting through their deposits and savings, while reducing spending on basics like food or clothing. This finding can expand on previous findings regarding the relationship between FT and the use of financial coping strategies [9]. It would be important for oncology professionals to assess how financial burden force patients to change their lives as above. Of note, any financial status was not significantly associated with FT in multiple regression analysis. Participants might have to worry about future payment even if they had more income and/or less expenditure. Worry about financial problems differ from actual financial problems, such as inability to pay healthcare costs [20]. Our findings suggest that FT in this population could heighten due to financial worry rather than due to a direct impact of financial burden. Worry about affording healthcare predicts more cost-related non-adherence [21]. Similarly, in this study, financial hardship put participants at a crossroads regarding whether they should continue cancer treatment, thus supporting the importance of intervention to FT caused by molecular-targeted and immune therapy.

Our results also suggest that FT might become severe as the impact of financial burden on patient experiences spread, and vice versa. Participants with higher FT reduced spending on basics like food or clothing, and further made up for shortage of funds for treatment expenses by cutting through their deposits and savings, which resulted in hesitating continuing treatment based on finances. In fact, participants’ FT was significantly related to the number of patient experiences associated with the financial burden. Patients would experience profound FT as a result of the accumulation of diverse experiences associated with financial burden. This finding is important in that it confirms that multifaceted aspects of experience associated with the financial burden of cancer treatment can cause FT. Material conditions (financial spending and financial resources), psychosocial responses (affect), and coping behaviors (support seeking, coping care, and coping lifestyle) have been proposed as conceptual domains and subdomains for subjective financial distress in patients with cancer [3]. Our results are consistent with the conceptual framework and suggest that participants experience profound FT not only from the material use of their deposits and savings but also from psychosocial and behavioral coping, owing to continuous payments for treatment. Oncology professionals must have a multifaceted understanding of the financial burden experienced by patients.

This study had some limitations. First, this study was an observational study without control (e.g., cancer patients who received cytotoxic chemotherapy). We could not refer to characteristics of FT in molecular-targeted and immune therapy in comparison with that in other cancer treatments. Also, we recruited participants irrespective of the price of the drug that they used. Second, the participants were recruited by convenience sampling and were small in number. Selection bias cannot be thus avoided. Moreover, the age restriction of the inclusion criteria limits the generalizability of the findings. Third, this was a cross-sectional study, hence we could not identify the causal relationship between FT and patient experience. The reverse possibility thus needs to be considered. Fourth, the participants’ responses to financial burden might have been influenced by recall bias because they were asked to respond to the period since the start of the cancer treatment they were receiving. The participants evaluated the experience for a median of 11 months. Finally, we could not consider the impact of other factors regarding disease and treatment. Notably, physical conditions, such as types of cancer, disease status, and symptoms related to cancer and its treatment, need to be considered because the presence of comorbidities was a significant factor of FT. Despite these limitations, the findings of this study revealed the importance of focusing on patient experiences to consider the financial aspect of molecular-targeted and immune therapies. Multicenter recruitment and a high response rate are the strengths of this study.

In conclusion, patients receiving molecular-targeted and immune therapies for cancer are at risk of experiencing profound FT, leading to a reduced quality of life. Profound FT can be caused by multifaceted experiences associated with the financial burden of cancer treatment. The financial burden of continuing molecular-targeted and immune therapies can even make patients hesitant to continue cancer treatment. Oncology professionals, including physicians, nurses, pharmacists, medical affairs, and social workers, need to consider multifaceted aspects of patient experience linked to financial burden and screen financial worry before actual financial problems appear. The independently related factors that we identified have the potential to serve as indicators of profound FT and the need for specialized intervention.
